# A multi-centre pilot study of iodine status in UK schoolchildren, aged 8–10 years

**DOI:** 10.1007/s00394-015-1014-y

**Published:** 2015-08-15

**Authors:** Sarah C. Bath, Emilie Combet, Patrick Scully, Michael B. Zimmermann, Katharine H. C. Hampshire-Jones, Margaret P. Rayman

**Affiliations:** 1 Department of Nutritional Sciences, Faculty of Health and Medical Sciences, University of Surrey, Guildford, Surrey GU2 7XH UK; 2 Human Nutrition, School of Medicine, College of Medical Veterinary and Life Sciences, University of Glasgow, Glasgow, G31 2ER UK; 3 Human Nutrition Laboratory, Swiss Federal Institute of Technology (ETH), Zurich, Switzerland

**Keywords:** Iodine, UK, Children, Diet

## Abstract

**Purpose:**

Iodine, as an essential constituent of thyroid hormones, is required for brain development. Iodine status is low in some UK population groups, notably in teenage girls, women of childbearing age and pregnant women. We aimed to assess iodine status of UK schoolchildren as there are no data on children below 14 years of age.

**Methods:**

Children (boys and girls) aged 8–10 years were recruited to a cross-sectional study from schools in three areas of the UK (Omagh, Northern Ireland; Glasgow, Scotland, and Guildford, South-East England). Spot urine samples, for measurement of urinary iodine concentration, were collected in the winter months (November 2012 to March 2013) and in the summer, in Omagh only (September 2013). A food frequency questionnaire was completed.

**Results:**

A total of 168 schoolchildren provided 165 urine samples. The median urinary iodine concentration was 161 µg/L in winter samples (*n* = 134) and 127 µg/L in summer samples (*n* = 31). The median urinary iodine concentration for the whole group was 144 µg/L, weighted to account for the unequal proportion of samples from the two seasons. The children are classified as iodine-sufficient by WHO criteria (100–199 µg/L), even in the summer. Milk intake was positively associated with iodine status.

**Conclusions:**

This pilot study suggests that iodine deficiency is unlikely to be a problem in UK children aged 8–10 years. This could be a result of higher intake of milk, the principal UK dietary iodine source, in this age group than in teenagers and adults. Further assessment of iodine status in a representative sample of UK schoolchildren is required.

## Introduction

Iodine deficiency in a population is of public health concern as iodine, required for thyroid hormone (T4 and T3) production, is essential for brain and neurological development [1]. Iodine deficiency during pregnancy, infancy and childhood may have long-term implications for neurodevelopmental outcomes (e.g., cognition), as rapid brain development occurs during these periods [2]. Severe iodine deficiency is associated with goitre and cretinism [1], while even mild-to-moderate iodine deficiency during pregnancy has been associated with poorer scores for IQ, reading [3] and spelling [4]. There is also evidence that iodine supplementation may benefit older children (10–13 years) in regions of mild-to-moderate iodine deficiency as two randomised, placebo-controlled, trials (RCTs) demonstrated improvements in some cognitive scores [5, 6].

The UK iodine Reference Nutrient Intake (RNI) for children aged 7–10 years is 110 μg/day [7], while the World Health Organisation (WHO) recommendation for 7- to 12-year-olds is 120 μg iodine per day [8]. For assessment of populations, the WHO recommends that spot urine samples are collected and urinary iodine concentration (UIC) is measured as it is reflective of recent iodine intake [8]. The median UIC is then compared to the criterion for iodine adequacy in a population, which is 100–199 μg/L for school-aged children [8].

Goitre was endemic in the UK in the nineteenth and early twentieth century, but was largely eradicated from the 1960s onwards [9]. This led to the general belief that the UK was iodine-sufficient and, until 2011, the UK was one of a few European countries that had no recent data on population iodine status (as assessed by urinary iodine concentration). However, a UK nationwide survey of over 700 schoolgirls, aged 14–15 years published in 2011 [10], revealed mild iodine deficiency (median 80.1 μg/L) since which time the UK has been classified as a mildly iodine-deficient country [11].

The eradication of goitre in the UK was driven by both increased milk iodine concentration (as a result of iodine supplementation of livestock that began in the 1930s to improve reproductive performance and milk yield) and increased milk consumption in the post-war years [9]. Milk iodine concentration is relatively high in the UK (mean 300 μg/L) [12], and milk and dairy products are still the major source of iodine in the UK diet, contributing up to 33 % of intake in adults and 51 % of intake in children aged 4–10 years, according to recent data from the National Diet and Nutrition Survey (2008–2012) [13]. However, there is a large seasonal variation in milk iodine content such that the concentration in winter milk is approximately double that of summer milk [12, 14]; this has a knock-on effect on iodine status, with higher iodine intake and excretion in the winter months [10].

Although there are UK data that suggest iodine deficiency in women of childbearing age and pregnant women [15], there are no data on iodine status of children below the age of 14 years, despite the WHO recommendation that population iodine status should be assessed in school-aged children (6–12 years) [8]. The current study therefore aimed to collect data on iodine status and intake of iodine-rich foods in children aged 8–10 years from three regions of the UK: South-East England, Scotland and Northern Ireland. On the basis of data in other UK age groups, we hypothesised that children would be iodine deficient.

## Methods

### Subjects

Children were eligible for inclusion in the study if they were aged 8–10 years and were not taking medication for thyroid disease or using an iodine-containing supplement.

Children were recruited from three centres in the UK: Northern Ireland (Omagh), Scotland (Glasgow) and South-East England (Guildford) during the winter months (November 2012 to March 2013). These locations were chosen for the following reasons: Glasgow and Omagh as representative of the highest (Scotland) and lowest (Northern Ireland) iodine status regions in the Vanderpump et al. [10] study of UK teenage girls, while Guildford in South-East England was chosen as data on iodine status of women of childbearing age [16] and pregnant women [17] have already been collected in this area. Schools that were willing to participate in the study were selected from the local areas; one school in Guildford, two schools in Glasgow and three schools in Omagh were used to ensure the minimum sample size per centre (*n* = 30) was reached. In each school, a presentation was given during school assembly by one of the study team members; the purpose of this was to explain the research and protocol to the children and teachers but researchers refrained from providing information on dietary sources of iodine during the presentation.

Iodine status is known to vary by season [10], and we chose to recruit in the winter as a best case scenario—if iodine deficiency was found when milk iodine content was at its peak, deficiency would also be likely in the summer. However, the opportunity to collect samples from children in the summer months in Northern Ireland arose as part of an M.Sc. project and therefore 36 children were recruited in September 2013 (from two of the schools used in the winter cohort and an additional school) according to the same protocol as for the winter cohort. Three children in the summer cohort were excluded from analysis since they fell outside the age inclusion criterion.

The study was conducted according to the guidelines laid down in the Declaration of Helsinki, and all procedures involving human subjects were approved by the University of Surrey Ethics Committee (EC/2012/106/FHMS). As the research involved children under the age of 16 years, parents or guardians gave their written, informed, consent for the child to participate in the study.

### Procedure

Study packs were distributed to the children on a Friday afternoon to take home over the weekend. These packs included a detailed information sheet for the parents (with contact details of the study team to enable parents to ask questions) and the consent form. The packs also contained an information sheet written for the child and an assent form that the child could complete. Parents or guardians completed a Food Frequency Questionnaire (FFQ) on behalf of their child and provided details on the child’s age and sex.

The children were required to collect a urine sample before school on the Monday morning into the wide-neck 125-mL polypropylene urine container that was provided in the study pack; we do not have information on the exact time and nature of the urine collection (i.e. whether it was the first void of the day or a fasted sample). All completed paperwork and the urine sample were then brought to the school where the packs were collected by a member of the study team. All urine samples were kept frozen (minimum −20 °C) until analysis.

### Analysis of the food frequency questionnaire

The 23-item FFQ was designed primarily to capture information on iodine-rich foods (milk, dairy products, iodised salt, eggs and seafood), i.e. those previously identified as important contributors to iodine status in UK studies [10, 16–18]. However, it also included some questions on other food groups (i.e. fruit and vegetables, meat and poultry, starchy foods and sugary foods) to disguise which foods were iodine rich in case parents might be tempted to alter their child’s diet prior to urine collection; these additional food items were not analysed in relation to urinary iodine excretion.

There were six daily frequency options for milk, as used in our previous research on pregnant women, ranging from “none” to “more than 570 mL” [17, 18]. For weekly egg consumption, the options were “none”, “one or two”, “three to six” and “more than six” per week. The FFQ also gathered data on the type of milk consumed (i.e. cows’, goats’, sheep’s milk or soya drink) and whether organic milk was regularly used, as organic milk is known to have a lower iodine concentration than conventional milk [14]. The FFQ also asked if iodised salt was used (yes/no). This question reflected use of iodised table salt (i.e. domestic use) and was not intended to capture information on iodised salt intake from processed foods; there is minimal use of iodised salt in food manufacturer in the UK and no national iodised salt policy [19]. For all other food items, the FFQ design was based on that of the ALSPAC FFQ for children [20] and had five frequency options. The responses were assigned codes to represent weekly frequencies as follows: never/rarely = 0; once a fortnight = 0.5; one to three times/week = 2; four to seven times/week = 5.5; once a day or more = 10 [20]. If more than one frequency option had been ticked, the lowest frequency was chosen; food items with missing entries (seven food items had up to two missing entries) were coded to “never or rarely”.

For the purposes of statistical analyses, daily milk consumption was recoded as follows: (1) <140 mL, (2) 140–280 mL, (3) 280–425 mL, and (4) >425 mL; children who reported consuming soya or rice drinks were recoded to “never” for daily milk consumption so that this variable only reflected dairy milk intake. Since there were no subjects in the “more than six” category, egg consumption was collapsed into three categories (1) “none”, (2) “one or two”, and (3) “three to six” per week. Responses for individual dairy products (cream, yoghurts, dairy desserts, butter and cheese) and fish (white fish, oily fish and shellfish) were summed and recoded to reflect high and low intake (i.e. above or below the median). For seafood consumption, an additional category of “non-consumer” was included, which was not possible for dairy products as there were no non-consumers.

### Laboratory analysis

All urine samples were shipped (on dry ice) to ETH Zürich for analysis. Urinary iodine concentration was measured in duplicate using a modification of the Sandell-Kolthoff reaction with spectrophotometric detection [21]. The coefficient of variation for UIC (±SD) in the ETH laboratory is 11.5 % at 31 ± 4 μg/L and 3.6 % at 212 ± 8 g μg/L. The ETH iodine laboratory participates successfully in the quality-assurance programme of the Centre for Disease Control, entitled “Ensuring the Quality of Urinary Iodine Procedures” (EQUIP) [22], using certified reference materials to ensure the accuracy of the method. Urinary creatinine concentration was measured in the biochemistry laboratory at the University Hospital, Zürich, by the Jaffe rate method.

### Iodine status

The iodine status of the group was described by comparing the median UIC value with the WHO UIC cut-off for iodine adequacy in school-aged children [8]. Using urinary creatinine concentration in a spot urine sample can correct UIC for intra-individual variation in daily urine volume produced. We therefore present data as both UIC and the iodine-to-creatinine ratio.

### Statistical analysis

Urinary iodine concentration and the iodine-to-creatinine ratio were not normally distributed and therefore medians with the 25th and 75th percentiles are reported. To calculate a median for the whole group, cases were weighted to account for the fact that the proportion of samples from the summer and winter seasons were unequal (18.8 and 81.2 %, respectively); an expected proportion of 50 % in each season was used. UIC and the iodine-to-creatinine ratio were log-transformed using the natural logarithm to allow parametric testing.

As a result of the known seasonal variation in iodine content of milk and dairy products [9, 12], and the fact that the summer cohort was recruited from just one UK centre, the summer and winter cohorts were not combined for analysis of the relationship between iodine status and dietary intake. As there was only a small number of children in the summer cohort, analysis of the FFQ and effect of age and sex of the child was restricted to those recruited in the winter months (*n* = 133).

A General Linear Model was constructed using either (log) UIC or iodine-to-creatinine ratio as the dependent variable with the dietary variables (frequency of milk, dairy products, seafood, eggs and iodised salt consumption), age, gender and UK location entered as independent variables. The residuals of the model were checked for normality and homogeneity of variances was checked to ensure that these assumptions were not violated.

Significance was set at *p* < 0.05 and analyses were conducted using the Statistical Package for Social Sciences (version 21.0; SPSS, Inc., Chicago, USA).

## Results

A total of 168 children participated in the study and 165 provided a urine sample of which 134 (81.2 %) were in winter and 31 (18.8 %) in summer. Of the 135 children recruited in the winter (52 from Guildford; 53 from Glasgow; and 30 from Omagh), one child just returned a questionnaire, and 132 children provided both urine and questionnaire data. Of the 33 eligible children recruited in the summer (all from Omagh), 31 children provided both a urine sample and questionnaire data.

The subject characteristics for each season are shown in Table 1. There was no significant difference in the percentage of boys and girls or the proportion of children in each age category (i.e. 8, 9 or 10 years) between the three UK centres (*p* = 0.27 and *p* = 0.09, respectively). There was also no significant difference in milk (*p* = 0.23), seafood (*p* = 0.29), dairy product (*p* = 0.56) or egg (*p* = 0.27) consumption between centres.

**Table 1:** Subject characteristics for the winter and summer cohorts

	Winter (*n* = 133)^a^	Summer (*n* = 33)
Age (years)		
8	37 (27)	15 (42)
9	53 (39)	8 (22)
10	43 (32)	10 (28)
Sex		
Boys	68 (51)	20 (61)
Girls	65 (49)	13 (39)

Figures are *n* (%)

^a^Two children did not return the questionnaire

### Iodine status

The urinary iodine concentration and iodine-to-creatinine ratio are summarised in Table 2. Based on the overall (weighted) median UIC of 144 µg/L and the fact that just 4.2 % (*n* = 7) had a UIC below 50 µg/L, the group is classified as having adequate iodine status according to WHO/UNICEF/ICCIDD criteria [8]. UIC was below 100 µg/L in 40 samples (24 %) and above 300 µg/L in 23 samples (14 %).

**Table 2:** Urinary iodine concentration and urinary iodine-to-creatinine ratio, split by season of recruitment

Season	Location	Iodine concentration (µg/L)		Iodine-to-creatinine ratio(µg/g)	
		*n*	Median (IQR)	*n*	Median (IQR)
Both seasons	All^a^	165	144 (95–223)	164^b^	150 (102–202)
Winter	All	134	161 (105–253)	133^b^	157 (101–228)
	SE England	51	142 (85–197)	51	134 (91–182)
	Scotland	53	171 (131–254)	52^b^	188 (123–271)
	N Ireland	30	196 (111–347)	30	142 (85–255)
Summer	N Ireland	31	127 (93–164)	31	143 (102–193)

^a^Cases weighted to account for unequal proportion of summer and winter samples (18.8 and 81.2 %, respectively)

^b^Insufficient urine volume for measurement of urinary creatinine concentration in one sample

The samples collected during the summer (*n* = 31) had lower median UIC (127 vs. 161 µg/L) and iodine-to-creatinine ratio (143 vs. 157 µg/g) than those collected in the winter (*n* = 134; Table 2); when comparing summer and winter samples from Northern Ireland, the difference was significant for UIC (*p* = 0.03) but not for the iodine-to-creatinine ratio (*p* = 0.65). Nevertheless, the median UIC was above the cut-off for iodine adequacy (100 µg/L) even in the summer months. In the summer cohort, 33 % of children (*n* = 11) had a UIC below 100 µg/L compared to 22 % (*n* = 29) in the winter cohort.

**Fig. 1:**
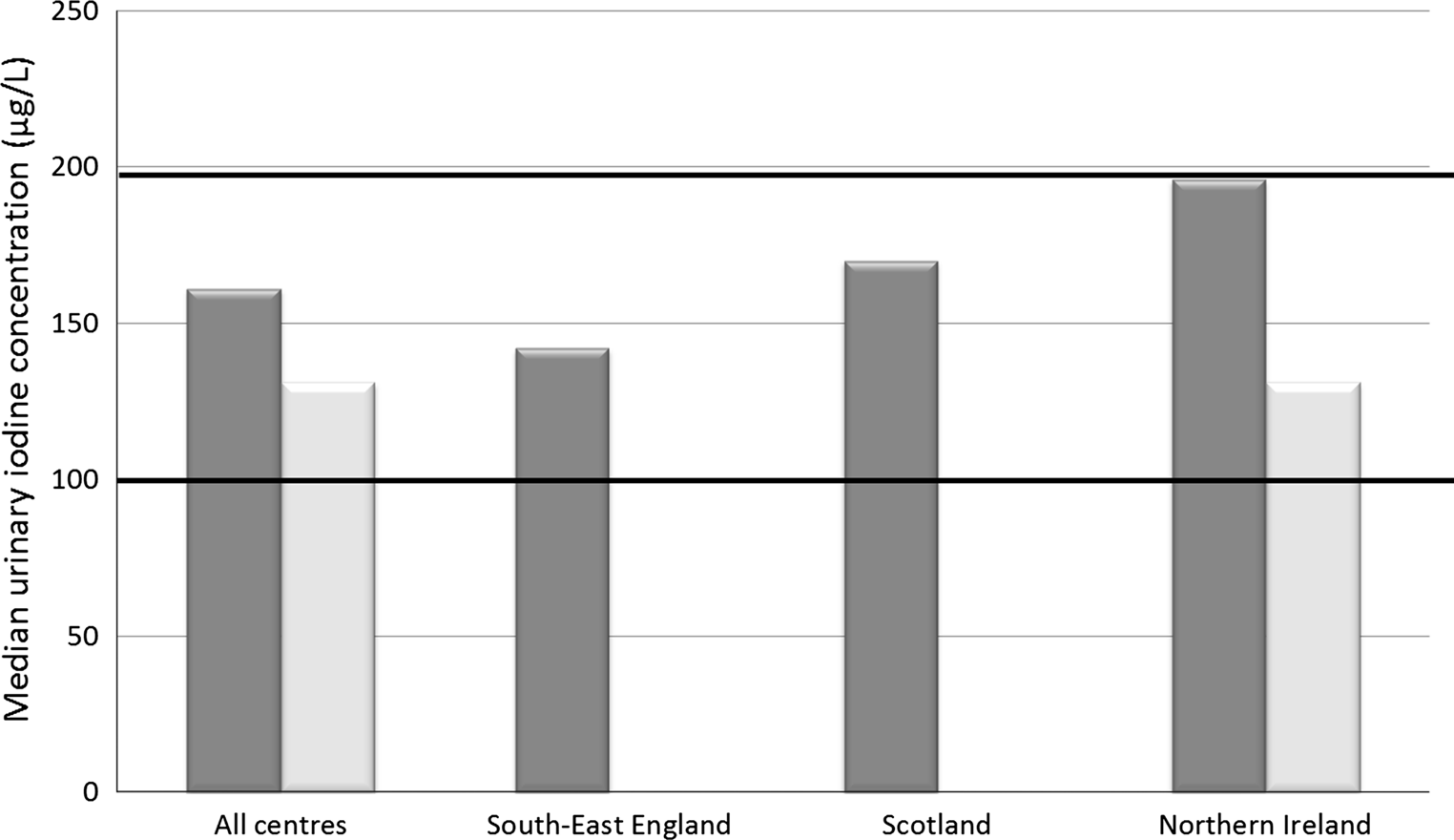
Urinary iodine concentration of UK schoolchildren (8–10 years); *dark* and *pale bars* represent results from samples collected in the winter and summer seasons, respectively. *Black horizontal bars* illustrate the lower and upper limits of the WHO adequate range for median iodine concentration in spot urine samples from a population of schoolchildren (100–199 µg/L) [8]

In the winter cohort, there was a significant difference in UIC (*p* = 0.04) and iodine-to-creatinine ratio (*p* = 0.009) between UK centres, although the pattern was not consistent between the two measures of iodine status; children in Northern Ireland had a higher UIC than the other centres, whereas those in Scotland had a higher iodine-to-creatinine ratio (Table 2; Fig. 1).

Neither UIC nor the iodine-to-creatinine ratio was significantly associated with age in the samples collected during the winter (*p* = 0.71 and *p* = 0.69, respectively). In winter samples, the median UIC was not significantly different in girls and boys (157 vs. 169 μg/L respectively, *p* = 0.81), nor was the median iodine-to-creatinine ratio (152 vs. 163 μg/g, respectively, *p* = 0.70). The number of samples collected in the summer was too small to stratify iodine results by age or sex.

### Relationship between diet and iodine status in the winter cohort

The relationship between dietary intake and urinary iodine excretion was explored for the 132 children who provided both a urine sample and questionnaire data (winter cohort only; Table 3).

**Table 3:** Unadjusted associations between dietary habits and urinary iodine excretion (iodine concentration and iodine-to-creatinine ratio); analysis restricted to the winter cohort only

Food group	Category	Urinary iodine concentration (µg/L)			Iodine-to-creatinine ratio (µg/g)		
		*N* (%)	Median (IQR)	*P* ^a^	*N* (%)	Median (IQR)	*P* ^a^
Milk (mL/day)^b^	<140	37 (28.0)	134 (87–164)	0.006	37 (28.2)	117 (74–173)	0.005
	140–280	46 (34.8)	165 (100–282)		46 (35.1)	195 (117–276)	
	280–425	24 (18.2)	180 (143–264)		23 (17.6)	150 (112–238)	
	>425	25 (18.9)	227 (146–286)		25 (19.1)	168 (129–216)	
Dairy products (freq/week)	<14	67 (50.8)	154 (102–264)	0.90	66 (50.4)	164 (111–256)	0.21
	≥14	65 (49.2)	163 (129–237)		65 (49.6)	149 (100–213)	
Eggs (number/week)	None	23 (17.4)	147 (94–253)	0.98	23 (17.6)	124 (108–206)	0.37
	One/Two	85 (64.4)	163 (109–244)		84 (64.1)	165 (111–225)	
	Three-six	24 (18.2)	176 (109–254)		24 (18.3)	177 (78–265)	
Seafood (times/week)	None	6 (4.5)	102 (34–172)	0.14	6 (4.6)	118 (98–184)	0.49
	≤2	58 (43.9)	168 (13–272)		57 (43.5)	139 (92–216)	
	>2	68 (51.5)	157 (102–227)		68 (51.9)	166 (111–246)	
Consumer of iodised salt	Yes	17 (12.9)	146 (83–287)	0.52	16 (12.2)	165 (78–237)	0.99
	No	115 (87.1)	163 (116–245)		115 (87.8)	162 (109–227)	

^a^Results from independent *t* test (dairy products and iodised salt) or ANOVA on the log-transformed UIC or iodine-to-creatinine ratio data

^b^Consumers of soya and rice drinks recoded to “none” for dairy milk intake

In univariate analyses, milk intake was positively associated with both UIC and iodine-to-creatinine ratio (*p* = 0.006 and *p* = 0.005, respectively). Median UIC increased with higher milk consumption (Table 3) and a Bonferroni post hoc test revealed that children who consumed <140 mL per day had significantly lower UIC than those who consumed 140–280 mL (*p* = 0.03) and >425 mL (*p* = 0.008). Similarly those children consuming <140 mL had significantly lower iodine-to-creatinine ratio than those consuming 140–280 mL (*p* = 0.004); all other post hoc comparisons were non-significant.

The majority of the children consumed cows’ milk (96.2 %), two consumed goats’ milk (1.5 %), two consumed soya drinks (1.5 %) and one child consumed a rice drink (0.8 %). A one-way ANOVA showed that there was a significant difference in UIC between the type of milk, or milk substitute, consumed (*p* = 0.006) and the post hoc test revealed that soya and rice drink consumers (combined) had significantly lower iodine status than those who consumed cows’ milk (*p* = 0.02) or goats’ milk (*p* = 0.006). Organic milk was normally consumed by 14.4 % of children (*n* = 19) but their UIC or iodine-to-creatinine ratio was not significantly different to those of other children (*p* = 0.37 and 0.41, respectively).

There was no significant difference in either UIC or iodine-to-creatinine ratio between high and low consumption of other dairy products (*p* = 0.90 and *p* = 0.21, respectively) or between categories of egg consumption (*p* = 0.98 and *p* = 0.37 respectively). Though non-consumers of seafood had a lower iodine status than consumers (Table 3), the differences were not significant for either UIC (*p* = 0.14), or iodine-to-creatinine ratio (*p* = 0.49). Just 17 (12.9 %) children were consumers of iodised salt and their UIC and iodine-to-creatinine ratio was not significantly different to those of non-consumers.

We used a General Linear Model to explore the associations between diet (intake of milk, dairy products, eggs, seafood and iodised salt), age, gender and UK geographical location (three centres) and both UIC and iodine-to-creatinine ratio. The model explained only 12.1 % of the variation in UIC and 11.6 % of the variation in iodine-to-creatinine ratio. Both milk intake and seafood were significantly, and positively, associated with UIC (*p* = 0.007 and *p* = 0.005, respectively). No other potential predictors of UIC were significant in the model. By contrast, the only significant predictors of iodine-to-creatinine ratio were milk intake (*p* = 0.002) and UK centre (*p* = 0.01); post hoc analysis revealed that Glasgow had the highest iodine-to-creatinine ratio, which was significantly higher than in Guildford (*p* = 0.01) but not significantly higher than in Omagh (*p* = 0.09).

## Discussion

The results of this cross-sectional pilot study provide the first data for iodine status in UK children aged 8–10 years and, contrary to our hypothesis, suggest that this age group is iodine replete. These findings contrast with those of iodine deficiency in other UK population groups, namely teenage school-girls [10], pregnant women [17, 23–25] and women of child-bearing age [16, 26]. Our weighted median UIC from both winter and summer cohorts and from both boys and girls (144 μg/L) cannot easily be compared with the median UIC of teenage schoolgirls (80.1 μg/L) as that value was not weighted and most (72.8 %) of the girls were recruited in the summer [10] which would have resulted in a lower median UIC owing to the lower iodine content of summer milk [12]. However, when comparisons are restricted to samples collected from girls in the winter, our median value is 1.7 times higher than that of UK teenage girls (157 vs. 95.1 μg/L) [10], suggesting that the children in the current study have considerably higher iodine status than UK teenage girls.

The fact that the children in our study are iodine-sufficient may be the result of higher milk consumption in school-aged children than in adults and teenagers, who have been the subject of previous UK iodine surveys [10, 16, 17, 23–26]. Children in the present study reported a higher intake of milk than in our studies of pregnant women; 37 % of children consumed more than 280 mL milk per day, compared to 28 % of pregnant women in Surrey [17] and 33 % of pregnant women in Oxford [18]. This observation is supported by data from the most recent (2008/09–2011/12) National Diet and Nutrition Survey (NDNS) where reported daily milk intake (from food diary analysis) is higher in children aged 4–10 years (average 198 g/day) than those aged 11–18 (average 142 g/day) or in adults aged 19–64 years (average 136 g/day) [13].

Our results support the view that iodine status in schoolchildren should not be used as a proxy for all population groups within a country [27, 28], as has been recommended in the past [29]. This is particularly true in countries such as the UK where the main sources of iodine are milk and dairy produce which are consumed in high quantities by children but not necessarily by other age groups; children are not a representative group with respect to intake of iodine-rich foods and would therefore present a biased estimate of population status. There is evidence that milk consumption tends to decline with advancing age [30], such that milk will contribute less to total iodine intake in adults; thus adults who consume less milk will be more at risk of iodine deficiency than children in the same country. The UK would be classified as iodine-sufficient if the results of this study were used instead of those from the study of teenage girls [10]. However, this would mask the extent of deficiency in the UK, particularly in pregnant women and women of childbearing age, who are vulnerable to the effects of iodine deficiency. However, it is important to keep in mind the fact that the published cut-offs to assess iodine status in adults and pregnant women have not been validated and thus describing population status in these vulnerable groups is perhaps more challenging than in school-aged children, where more research has been conducted and the cut-offs are evidence-based [29].

It is important to highlight the fact that although our results suggest that UK school-aged children are iodine-sufficient, this does not detract from the potential public health implications of iodine deficiency in UK pregnant women [17, 23–25]. This is suggested by a recent Australian study [4] that found an association between iodine deficiency in pregnancy and poorer scores for spelling and grammar in the offspring at age 9, despite the fact that the children had grown up in an iodine-sufficient environment (they were born at the time of a local voluntary programme of iodine fortification of bread) [4]. During pregnancy, there are critical developmental windows in which iodine deficiency may lead to irreversible adverse brain effects that cannot be overcome by later iodine sufficiency. During childhood, cognitive impairment due to iodine deficiency appears to be at least partly reversible as shown in two randomised placebo-controlled trials in mild-to-moderate iodine-deficient school-aged children [5, 6], where iodine supplementation improved performance on some, though not all, cognitive tests. Hence iodine deficiency in UK pregnant women may have negative consequences on offspring cognition, even if UK children are ingesting adequate iodine, though further research in this area is required.

Our results showed that there were significant differences in iodine status between the three recruitment centres, but all had a median UIC within the optimum range as defined by WHO et al. [8]. In the nationwide study of iodine status in teenage schoolgirls, Vanderpump and colleagues found that the lowest UIC was in Northern Ireland (Belfast), whereas we found the opposite in our study which showed the highest UIC in Omagh, Northern Ireland. When using the iodine-to-creatinine ratio, children in Scotland had the highest median value which supports the finding by Vanderpump et al. of the highest UIC values being in Scotland. The discrepancy between the results by centre for UIC and iodine-to-creatinine ratio cannot be explained but may reflect variation as a result of the relatively small numbers of children in each centre (e.g., 30 children in Omagh); hence, the results for each centre should not be interpreted in isolation but should be used to contribute to the overall median for children aged 8–10 years in the UK.

Creatinine adjustment of UIC is not recommended by the WHO, owing to concerns that malnutrition results in low creatinine excretion and can thus mask iodine deficiency [8]. However, malnutrition is not likely to be a concern in the UK and we have previously found that use of the iodine-to-creatinine ratio (rather than UIC) in samples collected from pregnant women improves the ability to relate iodine status either to child outcomes [3] or dietary intake [17, 18]. In the present study, it was not clear that creatinine adjustment improved the relationship with dietary intake. As we did not collect 24-h urine samples, it is difficult to estimate which measure is preferable in the estimate of iodine status in this UK age group. In adults there are published reference values for the expected 24-h urinary creatinine excretion according to age and sex of the individual, enabling estimation of 24-h iodine excretion, which has been shown to be superior to UIC and the crude iodine-to-creatinine ratio [31–33]. However, as children are still growing, which affects muscle mass and thus creatinine excretion [34], information in addition to age and sex may be required for more accurate use of the iodine-to-creatinine ratio; published data for expected 24-h creatinine excretion in children require anthropometric measurements (height and/or weight) [35], and unfortunately, these data were not available for this cohort. Therefore, we present results for both measures of iodine status but further study is required to evaluate the usefulness of the iodine-to-creatinine ratio in well-nourished children.

As in other UK studies [10, 16, 17], and those in Europe [36], milk intake was positively associated with iodine status, even after adjustment for other factors. The UIC of consumers of soya/rice drinks (*n* = 3) was significantly lower than that of cows’ or goats’ milk drinkers. Most soya and rice drinks available in the UK are not fortified with iodine and therefore are a poor source of iodine [37]. Children who regularly drink soya milk in preference to cows’ milk may be at risk of iodine deficiency, but the small number of consumers in this cohort means that firm conclusions cannot be drawn. Reflecting the high concentration of iodine in seafood [38], we found that non-consumers of seafood had lower iodine status than children who consumed some seafood, though this was only significant in the model for UIC and not for the iodine-to-creatinine ratio. Our results suggest that a diet that includes a regular intake of fish, milk and dairy products will help to ensure adequate iodine intake. Our study was too small to explore dietary patterns that may result in iodine deficiency, for example children who largely follow a milk-free diet.

The strengths of the present study include the fact that this is the first report of iodine status in this age group and has collected data from three regions of the UK, and in both seasons (for one location). However, we acknowledge that there are several limitations, including the fact that the sample size is relatively small (particularly for the summer season); however, according to the estimations by Andersen et al. [39], our overall sample size is above that required (*n* = 125) to estimate the iodine level in a population with 95 % confidence within a precision range of 10 %. Another limitation of the study is the fact that only spot urine samples were collected and while this is the WHO-recommended method for assessing population iodine status [8], it gives us no opportunity to explore whether the higher iodine status in this age group is as a result of lower total urine volume than in teenagers or adults, which has recently been suggested as a reason that age-specific cut-offs to indicate iodine sufficiency are required [40]. It would be interesting to compare the 24-h iodine excretion, as a hydration-independent measure, in UK children and women of childbearing age to evaluate whether the higher iodine status in children is confirmed after accounting for total urine volume.

In conclusion, our results suggest 8- to 10-year-old children in the UK have sufficient iodine intake, in contrast to recent findings from female teenagers and pregnant and non-pregnant women that suggest deficiency in these groups. This is a different situation to other countries, for example New Zealand, where iodine deficiency was demonstrated in children [41], adults [42] and pregnant women [43]. As a result of mild iodine deficiency, a national iodine fortification programme was introduced in New Zealand (and Australia) in 2009 [44]; if such a programme were to be introduced in the UK, careful consideration would need to be given to the effect that raising iodine status would have on children who are already iodine replete (by WHO criteria [8]). A recent Australian report details the change in urinary iodine concentration in children after introduction of the fortification programme but does not include information on effect on thyroid or other health outcomes in the children from regions with adequate iodine status at baseline [45]. In view of the relatively small number of children in our study, further investigation in a representative sample of UK schoolchildren is required before public health recommendations for the UK can be made.

## Abbreviations

EAR: Estimated average requirement
FFQ: Food frequency questionnaire
RNI: Reference nutrient intake
UIC: Urinary iodine concentration
WHO: World Health Organisation
